# The first long-read nuclear genome assembly of *Oryza australiensis*, a wild rice from northern Australia

**DOI:** 10.1038/s41598-022-14893-5

**Published:** 2022-06-25

**Authors:** Aaron L. Phillips, Scott Ferguson, Nathan S. Watson-Haigh, Ashley W. Jones, Justin O. Borevitz, Rachel A. Burton, Brian J. Atwell

**Affiliations:** 1 Department of Food Science, University of Adelaide, Adelaide, SA Australia; 2 ARC Centre of Excellence in Plant Energy Biology, Adelaide, SA Australia; 3 Research School of Biology, Australian National University, Canberra, ACT Australia; 4 ARC Centre of Excellence in Plant Energy Biology, Canberra, ACT Australia; 5 South Australian Genomics Centre, University of Adelaide, Adelaide, SA Australia; 6 Australian Genome Research Facility, Victorian Comprehensive Cancer Centre, Melbourne, VIC Australia; 7 School of Natural Sciences, Macquarie University, Sydney, NSW Australia

**Keywords:** Molecular biology, Plant sciences

## Abstract

*Oryza australiensis* is a wild rice native to monsoonal northern Australia. The International *Oryza* Map Alignment Project emphasises its significance as the sole representative of the EE genome clade. Assembly of the *O. australiensis* genome has previously been challenging due to its high Long Terminal Repeat (LTR) retrotransposon (RT) content. Oxford Nanopore long reads were combined with Illumina short reads to generate a high-quality ~ 858 Mbp genome assembly within 850 contigs with 46× long read coverage. Reference-guided scaffolding increased genome contiguity, placing 88.2% of contigs into 12 pseudomolecules. After alignment to the *Oryza sativa* cv. Nipponbare genome, we observed several structural variations. PacBio Iso-Seq data were generated for five distinct tissues to improve the functional annotation of 34,587 protein-coding genes and 42,329 transcripts. We also report SNV numbers for three additional *O. australiensis* genotypes based on Illumina re-sequencing. Although genetic similarity reflected geographical separation, the density of SNVs also correlated with our previous report on variations in salinity tolerance. This genome re-confirms the genetic remoteness of the *O. australiensis* lineage within the *O. officinalis* genome complex. Assembly of a high-quality genome for *O. australiensis* provides an important resource for the discovery of critical genes involved in development and stress tolerance.

## Introduction

The genus *Oryza* is made up of ~ 27 species that have been classified into 11 genome groups^1,2^. Two of these species (*O. sativa* and *Oryza glaberrima*), both of which belong to the AA genome clade, were independently domesticated in Asia and Africa, respectively. Together, these two domestic rice species serve as a staple food source for about 60% of the global human population. The remaining ~ 25 species occur in wild populations and have become specialised to occupy a diverse set of environments around the world^3^.

To date, the species and cultivars that make up the AA genome clade have provided most of the genetic variation and resources that have led to the success of domesticated rice^4^, while other genome clades have contributed relatively little to domestication. Encouragingly, rich diversity is apparent, even within landraces of just one *O. sativa* subspecies. For example, in a GWAS study for 14 agronomic traits (including tolerance to drought and degree of seed shattering) in 517 *indica* sub-species landraces, Huang et al.^5^ revealed ~ 3.6 million SNPs. Currently nine of the 11 *Oryza* genome assemblies available on Ensembl Plants; (accessed 26/2/2021)^6^ belong to the AA genome clade, one belongs to the BB genome (*Oryza punctata*), and one to the FF genome (*Oryza brachyantha*). Therefore, wild species of rice occupy a number of genomic clades that are not yet represented by an assembly and undoubtedly include traits that would be desirable in domestic cultivars. The wild species therefore represent vast reservoirs of untapped genetic variation that could be harnessed for domestic rice improvement^2–4,7^. Recently, Hiromi et al.^8^ provided an update to the OryzaGenome database which includes short-read sequencing data for several non-AA genome *Oryza* species. The availability of *Oryza* ‘-omics’ resources is increasing, yet the number of whole-genome assemblies for non-AA genome wild species remains low. Whole-genome assemblies of wild *Oryza* species will provide new sources of genes and gene variants/haplotypes, enhancing the ability of breeding programs to introduce desirable traits into domestic cultivars. However care is necessary to navigate issues such as linkage drag and suppressed recombination frequencies that may be associated with use of wild germplasm. Additionally, such projects could lead to the domestication of wild rice populations (e.g., through the targeted modification of domestication-associated traits, such as seed shattering)^9^. Overall, harnessing the genetics of wild *Oryza* species has wide application for cereal crop improvement and specifically for crop resilience. In fact, the *Oryza* Map Alignment Project (OMAP) and the International OMAP (IOMAP) recognised the value that the genome assemblies of wild *Oryza* species would provide to research in abiotic and biotic stress tolerance, and domestication, as long ago as 2003^10–12^. These projects recommended sequencing the genomes of several wild rice species but progress in this respect has been slow.

Recently, several non-AA genetic resources have become available, including genomes within the *O. officinalis* complex^13^ and the genome of *Oryza granulata*^14^. *O. granulata,* an upland wild rice species, has a demonstrated ability to tolerate drought stress and bacterial blight, providing a valuable new source of *Oryza* genetic variation. However, owing to its large genome size, high repeat content, and being a short-read assembly, the *O. granulata* genome assembly is highly fragmented, reducing its utility. These same difficulties (i.e., genome size and repeat content) have been encountered during assembly of the EE genome, represented solely by *O. australiensis,* which is reported to be the largest diploid *Oryza* genome*.*

*Oryza australiensis* is a perennial wild relative of rice endemic to the tropical regions of northern Australia. Due to the extremely hot and sporadically dry environment in which *O. australiensis* exists, this species has been described as an extremophile^3^. *Oryza australiensis* is the sole member of the EE genome clade and its genome has been estimated to be 965 Mbp in size, more than double that of *O. sativa* ssp. *japonica*^15,16^. The size of the *O. australiensis* genome is the result of the expansion of long terminal repeat (LTR) retrotransposon (RT) families (e.g., *Gypsy* and *Copia*), which comprise an estimated 65% of the genome. In comparison, the genome of *O*. *sativa* is composed of approximately 10% LTR-RT^15^. It is uncertain what led to the rapid genome-wide expansion of these LTR-RT families. However, it has been noted that the expansion of LTR-RTs facilitated rapid adaptive genome evolution in the *Oryza* genus, leading to speciation events^17^. These same processes may also play a role in the evolution of biotic and abiotic stress tolerances. Heat tolerance has been well-characterised in *O. australiensis*^18^, and transformation of domestic rice with a key gene (Rubisco activase) involved in heat tolerance fortifies grain yield during episodes of heat^19^. Further, Yichie et al.^20^ identified population-specific tolerance to salinity stress in *O. australiensis* accessions, and Hamzelou et al.^21^ report on drought tolerance in this species. The unique anatomy and photosynthetic efficiency of *O. australiensis* leaves also make it an important species for understanding how rice photosynthesis can be augmented^22^. In terms of food quality, the grain of *O. australiensis* has unique pigmentation and starch composition, distinct from Asian species^23,24^, that could prove to be a profitable food item in and of itself.

A highly-quality reference genome for *O. australiensis* could reveal novel loci for abiotic stress tolerance and help shed light on the genome evolution of the *Oryza* genus. However, since the conception of OMAP in 2003^11^, and despite international sequencing efforts since^12^, no research group has successfully assembled a high-quality, chromosome-level genome for *O. australiensis.* A previous attempt to assemble the *O. australiensis* genome with short reads resulted in a highly fragmented assembly due to the genome size and high repeat content^25^. Efforts such as those by Joly-Lopez et al.^25^ highlight the difficulties involved in assembling highly contiguous plant genomes with significant repeat content^26^.

In this study, we report on the assembly of a reference-quality *O. australiensis* nuclear genome from Keep River (KR; Northern Territory, Australia) into 1956 contigs using Oxford Nanopore Technology (ONT) long-reads. The contigs were subsequently scaffolded into 12 pseudomolecules representing 12 chromosomes using the *O. sativa* Nipponbare genome assembly as a guide.

## Methods

### Growth conditions for plant material used for genome sequencing

**Figure 1:**
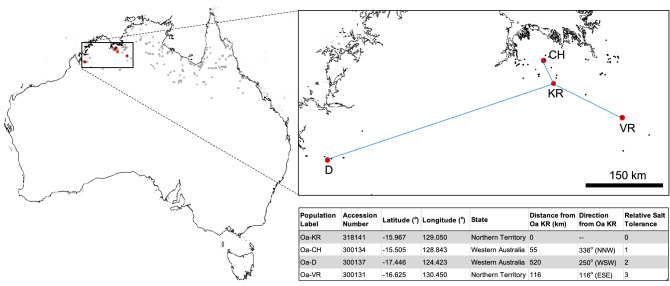
Locations of *O. australiensis* seed collection sites for this study. List of wild rice accessions (obtained from the Australian Grain Genebank) used in this study reported by Yichie et al.^19^ to demonstrate varying degrees of salt tolerance. Dots on the map show the occurrence of *O. australiensis* (retrieved from the Atlas of Living Australia). In-set table: accessions are ordered by salt tolerance, with Oa-KR being the most sensitive to salt, and Oa-VR being most tolerant^19^.

Seed of four *O. australiensis* populations was collected from the monsoonal savannah region of northern Australia (Fig. 1), compliant with the relevant national and international guidelines and legislation for wild species collections. All seed was obtained from the Australian Tropical Crops and Forages Collection (AusTRCF) of the Australian Grains Genebank (previously the Australian Plant Genetic Resource Information System) and as part of the Australian Tropical Crops Genetic Resource Centre Collection (ATCGRC). The Keep River accession used for the reference genome assembly bears the accession number AusTRCF 318143 and was collected by Dr I. Cowie at Darwin Herbarium, Northern Territory, Australia (Voucher ID# 9583) on 2nd May 2002 from a drying creek bed in the Keep River National Park after the passing of the summer monsoon. The other three accessions used for re-sequencing (CH, D and VR) are held in the same collection with accession numbers of AusTRCF 300134, 300137 and 300131. These lines are vouchered in the Jeff Corfield Collection in Townsville (Queensland, Australia) under the accession numbers JC 2317, JC 2336 and JC 2312, respectively. CH, D and VR were collected from the sites denoted in Fig. 1 between 1995 and 1997 by Mr I. Watson. Seed was stored at 4 °C until used. Seeds were imbibed in water for one hour and then surface sterilised in a 50% sodium hypochlorite bleach solution for 30 min followed by rinsing. Sterile seeds were sown onto absorbent cotton-lined petri dishes and incubated at 34 °C for 2 days to trigger germination. Germinated seeds were sown into a fine textured krasnozem soil (sourced from Robertson, NSW, Australia) and covered in a thin layer of vermiculite. Plants were grown in glasshouses at Macquarie University (Sydney, Australia) under a 30 °C/22 °C day/night cycle in April–June 2019.

### DNA extraction

Roughly 1 g of healthy, fully expanded leaf material was collected from ~ 2-month-old *O. australiensis* plants (one plant per genotype) and ground in a mortar and pestle under liquid nitrogen until a fine powder was achieved. High molecular-weight DNA was extracted at the Australian National University (ANU; Canberra, Australia) following the method of Jones et al.^27^.

### Nanopore library preparation, sequencing, and base calling

The sequencing library was prepared as per the manufacturer’s (Oxford Nanopore Technologies, Oxford, United Kingdom) instructions for genomic DNA by ligation method (SQK-LSK109) with small modifications. The manufacturer recommends using 0.2 pmol of DNA, however this is optimised for DNA fragments 8 kbp in length. As we were working with substantially longer (~ 20 kbp) DNA fragments, we loaded 3 μg (0.23 pmol) of DNA.

The library was run on FLO-MIN106 (R9.4.1 revD) flowcells as per the manufacturer’s instructions. To obtain a good level of coverage of the *O. australiensis KR* genome, the same library preparation for this sample was loaded onto three separate flow cells (i.e., the genomic DNA from one plant was sequenced on three flowcells). The raw signal intensities obtained from the flow cells were used by *guppy* (version 3.0.3) from ONT to perform base calling.

### Illumina sequencing

Aliquots (containing 2.24 ng DNA) of the same DNA samples from the four *O. australiensis* genotypes described above were prepared for Illumina short read sequencing. Short-read libraries were created using the Illumina Nextera tagment DNA enzyme (TDE1) according to Jones et al.^28^. Libraries were size selected for 350–600 bp fragments using a PippinHT (Sage Science). Sequencing was performed on an Illumina NovaSeq 6000 S4 flow cell 300 cycles (150 bp paired end), being multiplexed with other projects. Sequencing was performed at the Biomolecular Resource Facility, ANU.

### PacBio Iso-Seq library preparation and sequencing

Young grains were harvested during the milky period of development from mature *O. australiensis KR* plants from the same collection as the individual used for ONT and Illumina sequencing (i.e., different plants from the same population; germinated as above and grown in a glasshouse at 30 °C with ambient light at The Plant Accelerator, University of Adelaide, Adelaide, South Australia from February to June 2020). Some developing grain still possessed their pedicel/rachilla and pollen-containing anthers and these were included in the samples to increase the diversity of transcripts in the RNA library.

The growth zones of ten 115-day-old *O. australiensis KR* plants were excised and used for RNA extraction. The growth zone is defined here as ~ 10 mm from the base of the youngest leaves emanating from the central cylinder of the plants. It was necessary to remove surrounding leaf sheathes to access the central cylinder.

A separate cohort of *O. australiensis KR* plants was germinated as above, except germination was achieved with an incubation temperature of 30 °C. Germinated seeds were transferred to a growth rack partially submerged in a ½ Hoaglands solution (pH 5.01) spiked with 25 mg/L ferrous sulphate. After seven days of growth, root tips (5 mm from the end of the root), older seminal roots with young lateral roots, and healthy fully expanded leaves were harvested, snap frozen in liquid nitrogen and stored at − 80 °C. The growth solution was changed once during this time.

An additional cohort of *O. australiensis KR* seed was germinated at 30 °C and submerged in water in the dark to promote coleoptile growth. After six days under these conditions, etiolated coleoptiles were harvested as above.

Total RNA was extracted from the six tissues (7-day-old leaves, 7-day-old root tips, 7-day-old mature root sections, 6-day-old hypoxic coleoptiles, reproductive tissue, and 118-day-old growth zones) with the Sigma Spectrum Plant Total RNA Kit according to the manufacturer’s instructions, with minor modifications for the developing grain sample as per Betts et al.^29^. RNA samples were dried in RNA Stable tubes as per the manufacturer’s instructions and shipped to DNA Link (Seoul, South Korea). RNA quality and concentration of the samples were checked with a Nanodrop and with a Bioanalyzer 2100 instrument (Agilent, Santa Clara, USA). Only low-quality RNA (RIN < 7) could be extracted from the older seminal roots so this sample was omitted from further analyses.

RNA samples were barcoded upon conversion to cDNA libraries (to facilitate multiplexing) following the manufacturer’s instructions of the SMARTer PCR cDNA Synthesis kit. cDNA libraries were amplified by PCR and then an equal concentration of cDNA from each was pooled into a single library. The library was prepared for SMRTbell sequencing with the Express Template Prep 2.0 kit. Iso-Seq sequencing was carried out on a Sequel II PacBio instrument using a single SMRT cell.

Iso-Seq reads were processed through the IsoSeq v3 pipeline to generate high quality polished isoforms. Briefly, raw data is converted from multiple subreads per ZMW into a single consensus CCS read (minimum read quality of 0.9), demultiplexed and clustered (minimum predicted accuracy of 0.99). The aim of the clustering step is to collapse the CCS reads derived from different isoforms of the same gene or from the same transcript isoform but which differ slightly in their 5′ and 3′ ends, typically as a result of RNA degradation.

### Nuclear genome assembly and assessment

A summary of all reads obtained during this study can be found in Table 1. *Jellyfish* (RRID:SCR_005491)^30^ and *genomescope2* (RRID:SCR_017014)^31,32^ were used to estimate the size, unique sequence content, and heterozygosity of the *O. australiensis* KR genome using 17-mers in the KR Illumina short read libraries. Raw MinION reads for *O. australiensis KR* were corrected and then de novo assembled using *Canu* v1.9 (RRID:SCR_015880)^33^ as follows: canu -p oryza -d canu genomeSize = 965 m -nanopore-raw all.fastq.gz -gridOptions = "–time = 72:00:00" -obtovlThreads = 24 -batMemory = 9. The MinION long reads and the Illumina short reads were mapped to the resulting contigs using *minimap2* v2.17 (RRID:SCR_018550)^34^. Alignment files were used to polish the genome assembly with *HyPo* v1.0.3^35^. *JVarkit* was used to visualise long-read coverage of the contigs^36^. Contigs were checked for microbial contamination by querying BLAST-generated taxon ID numbers with the lineage command of *taxonkit* v0.6.0^37^. Contigs found to contain non-eukaryotic sequences were interrogated using the online genome browser IGV-Web (RRID:SCR_011793) by mapping long reads back to the assembly and checking the coverage and overlap of long reads with non-suspect sequences^38^. *Purge Haplotigs* (v1.1.2) was used to determine whether the wild rice genome assembly contained any haplotigs or junk sequences^39^.

**Table 1:** Summary of reads used for each step of genome assembly, polishing, variant detection and annotation.

Purpose	Sequencing platform	Sample	No. reads (Millions)	Mean Read Length (kbp)	Read N50 (kbp)	No. bases (Gbp)	Coverage (x)
Genome assembly and polishing	MinION FLO-MIN106 R9.4.1 revD	*O. australiensis*—KR 1	0.45	26.24	40.31	12	12
		*O. australiensis*—KR 2	0.94	23.22	35.14	22	23
		*O. australiensis*—KR 3	0.62	24.95	37.38	15	16
Genome polishing	Illumina NovaSeq	*O. australiensis*—KR	138.81	NA	NA	19	21
Genetic similarity	Illumina NovaSeq	*O. australiensis*—CH (300134)	143.27	NA	NA	20	22
		*O. australiensis*—D (300137)	87.27	NA	NA	12	13
		*O. australiensis*—VR (300131)	139.67	NA	NA	19	21
Genome annotation	PacBio Sequel II	*O. australiensis*—KR Leaf	0.24	2.69	NA	0.65	NA
		*O. australiensis*—KR Coleoptile	0.18	2.29	NA	0.42	NA
		*O. australiensis*—KR Root Tip	0.3	2.16	NA	0.64	NA
		*O. australiensis*—KR Growth Zone	0.3	2.75	NA	0.84	NA
		*O. australiensis*—KR Reproductive Tissue	0.24	2.46	NA	0.59	NA

See Fig. 1 for details on the accessions. KR 1, KR 2, and KR 3 are the reads obtained from a single genomic DNA preparation sequenced on three different flowcells. These reads were derived from the same *O. australiensis KR* plant and were used for the assembly of the reference *O. australiensis KR* genome. The same DNA preparation was used for the KR Illumina NovaSeq library preparation. CH, D, and VR (accession numbers appear in parentheses) refer to different accessions of *O. australiensis* that have been shown to vary in their tolerance to salt^20^. Reads from these accessions were used to estimate genetic similarity between the genotypes. Multiple *O. australiensis* KR plants were used for RNA extraction for Iso-Seq analysis.

The quality of the genome assembly was assessed by analysing the quality of the LTR-RT elements of the *O. australiensis KR* genome. LTR-RT elements are difficult to assemble. As such, LTR-RT assemblies provide a proxy for the quality of the rest of the genome. LTR regions were identified with *LTR_Finder_Parallel* v1.1 (RRID:SCR_018969)^40^ and *LTR_Harvest*^41^. The outputs were used as input for LTR_Retriever v2.8.7 (RRID:SCR_017623)—to generate LTR Assembly Index (LAI) scores^42^. The quality of the assembly was also assessed with *BUSCO* v4 (RRID:SCR_015008)^43^ using the *Poales* database (poales_odb10.2019-11-20; contains 4896 sequences) as the lineage. Contigs were mapped to the *O. sativa* Nipponbare reference genome (GCA_001433935.1) with *minimap2* and visualised with *MashMap* v2.0^44^.

Pseudomolecules for the assembly were generated using *RagTag* v1.0.0^45^ using version 7 of the *O. sativa* Nipponbare reference genome^46^ as a guide for the ordering and orientation of contigs into pseudomolecules (Supplementary File S1a). Contigs that could not be assigned to a pseudomolecule were concatenated (with 100 Ns at contig boundaries) into a ‘chromosome’ called ChrUn, though we used the unassigned contigs themselves for most of the downstream analyses. To understand why the unassigned contigs could not be placed into a pseudomolecule, the identity of a random subset of the unassigned contigs was determined by BLAST searches and the average LTR content of ChrUn was determined by *LTR_Retriever*. Contigs flagged as repeats, haplotigs or junk by *Purge Haplotigs* were kept in the assembly for completeness but were not included in scaffolding (see above). *JVarkit’*s *WGSplotter* was used to visualise long-read genome coverage^36^. BLAST was used to determine the identity of a random subset of the unassigned contigs. Subsequent annotation of the unassigned contigs (see below) also helped in determining the identity of the sequences that could not be assembled into pseudomolecules. The quality of the scaffolded assembly was assessed again with *BUSCO* and LAI scores, as above. *LTR_Retriever* was also used to produce estimates for LTR-RT insertion times. The quality of the assembly was further checked by mapping ONT long reads to the pseudomolecules to obtain a read mapping rate and to estimate read coverage across the assembly.

### Comparison of *O. australiensis* KR assembly to the *O. sativa* Nipponbare assembly

The *EDTA*-masked (see below) pseudomolecules were aligned to the Nipponbare genome using *minimap2* with the ‘asm20’ setting. The mapping file was used as input for *dotPlotly*^47^ to generate a dot plot of the whole-genome alignment with the following settings: “-m 2000 -q 500000 -k 12 -l -p 12”. The resulting plot was used to interrogate suspected structural variations (SVs) in the wild rice genome. We used *O. sativa* vs *O. punctata* Chr2 (CM002489.2; a BB genome rice), *O. sativa* vs *O. australiensis* KR Chr2, and *O. australiensis* KR vs *O. punctata* Chr2 alignments to inspect a region on Chr2 in these species which has been reported as an inversion in non-AA rice genome^1^. Alignments were made as described above, except “-m 5000” was used for *dotPlotly* visualisation. To check whether reference bias in the *O. australiensis* scaffolds was affecting the Chr2 SVs, we scaffolded the KEEP contigs using the *O. punctata* reference genome (AVCL0200000). We aligned the *O. punctata*-guided *O. australiensis* Chr2 to *O. punctata* Chr2, and then to *O. sativa* Nipponbare Chr2 as above.

To check whether the SVs were real, contig coordinates within the scaffolds were extracted from the *RagTag* AGP file and these were used to build a ‘cytoband’ file for *karyoploteR*^48^. The coordinates for the SVs (± 10 kbp) identified in the *O. australiensis KR* and *O. sativa* genome alignment were added to the ‘cytoband’ file, and these were used to visualise the boundaries between contigs and SV events using *karyoploteR*. Further, long reads were mapped to the *O. australiensis* KR scaffolds to check the level of coverage across SV junctions. SV junctions with low coverage were coloured red to identify them as suspicious.

To identify single nucleotide variations (SNVs) in the wild rice genome, *O. australiensis KR* Illumina short reads were mapped to the Nipponbare genome with *minimap2*. The resulting BAM file was used as input for the *SAMtools* (RRID:SCR_002105)^49^ sub-command *mpileup,* and *BCFtools* (RRID:SCR_005227) called SNV variants. *VCFtools* (RRID:SCR_001235)^50^ filtered low quality SNVs with low-read support (minQ = 30; minDP = 3). High quality SNVs were visualised using *karyoploteR* in RStudio with a window size of 70 kbp.

### Genome repeat annotation

*LTR_Retriever* (see above) was used to provide an estimate of LTR-RT elements in the *O. australiensis* KR contigs and scaffolds. Repetitive DNA elements were annotated using the Extensive de novo TE Annotator (*EDTA;* Supplementary File S2b)^51^. A manually curated rice repeat-element library provided by the developers of *EDTA* was used to identify repeats in the *O. australiensis KR* genome. A library containing *O. sativa* ssp. *japonica* transcript sequences including untranslated regions (UTRs) was downloaded from the IOMAP Genomes Database^52^ and used by *EDTA* to mask endogenous genes from its annotation pipelines. *EDTA* also identifies repeat sequences in the input genome that were not part of the repeat library used for interrogation and then constructs a repeat library specific to the input assembly. Total repeat content was also estimated and annotated using the *RepeatMasker*-based *Repeat Masking* tool included in OmicsBox version 2.0.10^53,54^. The custom *O. australiensis KR*-specific repeat library generated by *EDTA* was used as input for the RMBlast search engine, while other settings were left as default.

### Protein-coding gene annotation

Evidence-based gene-finding was carried out with the *OmicsBox* Gene Finding tool with *O. sativa* set as the most closely related species^53,54^. A protein evidence file containing protein sequences from 12 species/accessions of rice downloaded from the IOMAP Genomes Database^52^ and the Iso-Seq isoforms were used as evidence for gene finding. Genes were searched on both strands of the pseudomolecules and the unassigned contigs and alternative splice variants were allowed. The resulting gene coordinates were used as input for the built-in *Functional Gene Annotation* workflow. This workflow uses *RepeatMasker* to mask repeat elements in the genome assembly before carrying out gene prediction with *Augustus*. Then, both *Blast2GO* and *InterProScan* pipelines were applied to search for gene and protein matches for the identified genes. Annotations were further supplemented by searching orthologues of the identified genes/proteins using *EggNOG-mapper* in *OmicsBox*. Annotations for the contigs and scaffolds were exported as GFF3 files (Supplementary Files S3c, S4d). To check whether the GFF3 files contained TE gene annotations, we used a list of known plant TE families from the Atlas of Plant Transposable Elements^55^.

### *O. australiensis* genotype diversity and genetic similarity

To identify SNVs between four genotypes of *O. australiensis* with demonstrated differences in salt tolerance^20^, the *O. australiensis* CH, D, and VR Illumina short reads (Table 1) were mapped to the scaffolded *O. australiensis KR* genome assembly using *minimap2* and visualised as above. Keep River short reads were also mapped back against the Keep River genome to provide a baseline of SNVs. *Mpileup* was used to call variants between the *O. australiensis* KR genome assembly and the mapped reads. VCF files were filtered using bcftools such that only reads with a mapping quality > 30, and only SNVs with a depth of 5× were considered. The patterns of SNV density described here were visually inspected.

Genetic similarity between the *O. australiensis* genotypes was determined using the Illumina short reads for each genotype as input for *kWIP*^56^. *kWIP* uses a Weighted Inner Product of different k-mer hash values derived from *khmer* (RRID:SCR_001156)^57^ to reduce the effect that erroneous read data has on estimates of genetic similarity. The estimate of genetic similarity was also extended to the *Oryza* genus using publicly available Illumina short read data derived from genomic DNA samples (see Supplementary File S5e for the list of accessions).

## Results

### Genome assembly, making pseudomolecules and genome quality

To generate long-read sequence data for assembly, DNA from a single *O. australiensis* Keep River (KR) plant was extracted, purified, and sequenced on three ONT MinIOn flow cells (KR1, KR2, and KR3). Additionally, the long-read DNA extract was used to generate Illumina NovaSeq short read data for *O. australiensis* KR. DNA extraction and Illumina short-read sequencing was also performed on *O. australiensis* genotypes CH, D, and VR (Fig. 1, Table 1). We generated 49 Gbp (2.01 million reads) of ONT MinION long read data and 19 Gb (138.1 million reads) of Illumina short read data for the *O. australiensis* Keep River accession. Further, we generated 3.14 Gbp (1.25 million reads) of PacBio Iso-Seq data from five distinct tissue types (Table 1, Supplementary Fig. S1). Finally, we generated 20 Gbp, 12 Gbp, and 19 Gbp of Illumina short read data for three additional *O. australiensis* genotypes (CH, D, VR, respectively; Table 1).

K-mer analysis of *O. australiensis* KR short reads by *jellyfish* and *genomescope2* estimated the genome size to be ~ 822 Mbp. About 41.9% of the k-mers were unique, which suggested that the genome contained 58.1% repeat content. Heterozygosity was estimated to be 0.001%. Based on these results, we did not expect heterozygosity to have greatly affected contig assembly.

*Canu* assembled a genome containing 980 Mbp across 1956 contigs (Table 2). This represents 101.5% of the expected *O. australiensis* genome size. The contig N50 was 1.9 Mbp, the largest contig was 24.9 Mbp in length, while the smallest contig was 1324 bp in length. After polishing with *HyPo* the size of the assembly increased to 996 Mbp (103.2% of the expected genome size). We achieved an average coverage of 38 × and the contigs aligned well with the *O. sativa* Nipponbare reference genome (Supplementary Figs. S2, S3). *BUSCO* and LAI scores of 91.9% (complete) and 15.2, respectively, indicate that we have assembled a highly complete contigs. We identified 93 haplotigs, 124 junk contigs, 889 repeat contigs, and 850 primary contigs using *Purge Haplotigs* (Table 2). Thus, after purging the assembly of all but the primary contigs, the final assembly size was 859 Mbp (*cv*. 965 Mbp estimated by flow cytometry^15^, and 822 Mbp estimated by k-mer counting—see above).

**Table 2:** Genome assembly statistics for *O. australiensis* keep River.

Stage	Feature (unit)	Value
Long reads	Post-QC bases (Gbp)	49.2
	Estimated coverage (x)	51
	Expected genome size (Mbp)	965
Genome assembly (contigs)	Assembled genome size (Mbp)	996
	Long read coverage (x)	38
	No. contigs	1956
	Contig N50 (Mbp)	1.9
	Contig L50	114
	Contig N90 (kbp)	186.7
	Contig L90	799
	BUSCO score (100% = 4896)	91.9
	Whole-genome LAI	15.2
Genome scaffolds	Scaffolded genome size (Mbp)	860.9
	Long read coverage (x)	46
	Placed contigs	693
	Unplaced contigs	157
	Total length of placed contigs (including Ns; Mbp)	812
	Total length of unplaced contigs (Mbp)	46.9
	Gaps (Mbp)	2.1
	BUSCO score (100% = 4896)	97.5
	Whole-genome LAI score	17.6
*LTR_Retriever* (scaffolds)	LTR-RT content (Mbp)	518.2
	LTR-RT content (%)	60.2

Scaffolding resulted in the placement of 693 of the 850 primary contigs into one of 12 pseudomolecules and increased assembly size to ~ 861 Mbp. Pseudomolecules contained 81.5% of all assembled contigs (~ 812 Mbp of 996 Mbp) and long read coverage increased to 46×(Table 2, Supplementary Fig. S4, Supplementary File S1a). The remaining 157 contigs (46.9 Mbp) could not be placed into a pseudomolecule. Analysis of the unassigned contigs by *LTR_Retriever* showed that these sequences were largely made up of repeat sequences (~ 80% LTR-RT content, versus ~ 60% in the pseudomolecules), explaining why they could not be placed in the scaffolds. After scaffolding with KEEP contigs only, the *BUSCO* completeness score of the assembly was 97.5% and the LAI was 17.6. To further assess the quality of our scaffolded genome, we mapped ONT long-reads to the pseudomolecules, where 99.87% of ONT long-reads aligned to our genome with high quality (average MAPQ = 47).

After aligning our assembly long-reads (i.e., KR1, KR2, and KR3) to our assembled genome we observed several positions in the *O. australiensis* KR assembly that have low long-read coverage (< 20x, *cv*. 46× scaffold coverage; Supplementary Fig. S4). These regions predominantly occur on chromosome 4 and coincide with regions that are composed of smaller contigs (Supplementary Fig. S5). Additionally, chromosome 4 was constructed from a larger number of contigs than all other pseudomolecules (76 contigs compared to an average of 56 ± 9 contigs per pseudomolecule; Supplementary Fig. S5).

### Structural variations (SVs)

**Figure 2:**
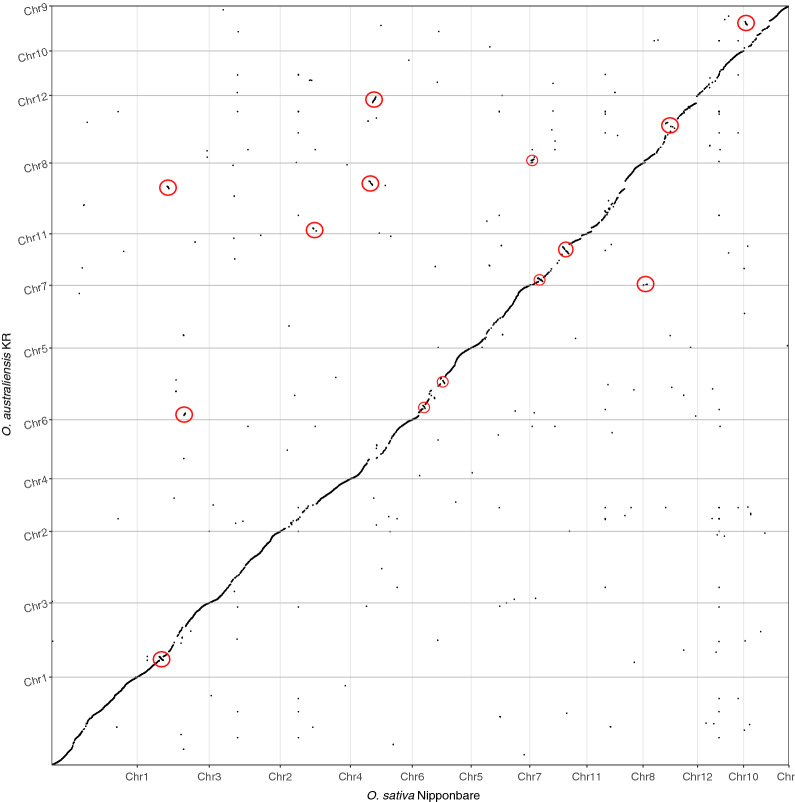
Alignment of scaffolded *O. australiensis* KR contigs (named Chr1–12) to the *O. sativa* Nipponbare reference genome. The wild rice genome was aligned to the domestic rice genome using *minimap2* and visualised using *dotplotly*. The 21 SVs that were investigated further are circled in red (some circles contain multiple SVs). Note: chromosomes do not appear in numerical order—*dotPlotly* orders the target sequence (*O. sativa*, here) by chromosome size; ChrUn is not included as it did not contain any large alignments.

We aligned the *O. australiensis* KR assembly to the *O. sativa* Nipponbare assembly and observed a high level of synteny between the two genomes (Fig. 2). We also observed 378 instances of non-syntenic alignment (e.g., sequence that occurs on *O. sativa* chromosome 1 aligning to *O. australiensis* KR pseudomolecule 3). Thus, the genome–genome alignment indicated the presence of SVs. We investigated 21 SVs further (8 translocations, 7 inverted translocations, 5 inversions, and 1 duplication). The largest SV detected was an inversion that occurred on Chr11 and was 2.95 Mbp long within the *O. sativa* Nipponbare genome and 7.2 Mbp long in the *O. australiensis* KR genome assembly. We show that some of the SVs are unlikely to be caused by scaffolding errors (Supplementary Fig. S5). Seven of the 21 reported SVs cross contig–contig boundaries. Further, by mapping the *O. australiensis* KR long reads to the pseudomolecules and observing continuous mapping of long reads at a depth of 31–75× coverage across SV junctions, we show that the investigated SVs are likely not artefacts of errors in the assembly of the contigs. All reported SVs require further investigation before they can be confirmed.

Recently, Stein et al.^1^ showed an inversion on chromosome two of non-AA rice genomes relative to AA genome rice. This inversion was not detected in the assembly presented here (Fig. 2). We verified the inversion reported by Stein et al.^1^ and the lack of inversion in the *O. australiensis* KR assembly by aligning *O. sativa* Nipponbare Chr2 with *O. punctata* Chr2 (Supplementary Fig. S6A) and *O. australiensis* KR Chr2 (Supplementary Fig. S6B), respectively. When we aligned *O. australiensis* KR Chr2 to *O. punctata* Chr2 we saw an inversion similar to the *O. sativa* Nipponbare vs *O. punctata* Chr2 alignment (Supplementary Fig. S6C). To check the validity of this finding, we mapped *O. australiensis* KR long reads back to the assembly and observed 38–43× coverage at the alleged SV junction. Further, the coordinates of the alleged SV (Chr2:74,761,274–74,941,712) in the *O. australiensis* KR assembly, derived from the alignment of *O. australiensis* KR Chr2 to *O. punctata* Chr2 (Supplementary Fig. S6C), sit well within the boundaries of a contig (tig00003125 occupies Chr2: 69,779,629–76,584,758). After aligning *O. punctata*-guided *O. australiensis* Chr2 to *O. punctata* Chr2 we again observed an inversion at these coordinates, but no inversion when *O. punctata*-guided *O. australiensis* Chr2 and *O. sativa* Nipponbare Chr2 (data not shown).

### Transcripts and genome annotation

Using a single PacBio Sequel II 8M SMRT Cell we were able to generate 3.3 million polymerase reads. Once these data were collapsed to Circular Consensus Sequencing (CCS) reads and demultiplexed, we had ~183–305 k CCS reads per tissue. Once the demultiplexed CCS reads were clustered we had ~21–35 k polished high quality (HQ) isoforms per tissue (Table 3). *BUSCO* analysis indicated that the Iso-Seq transcripts from the five selected tissues accounted for ~ 40% of the 4896 sequences in the *Poales* database. Only the HQ transcripts were used for annotation (low quality transcripts were discarded). A number of transcript isoforms were detected (see Supplementary Fig. S1B–D for examples of transcript isoforms for genes involved in stress response in rice).

**Table 3:** Summary statistics of the Pac Bio Iso-Seq data.

Tissue	# CCS reads	Mean length (kbp)	# Polished isoforms	
			High quality	Low quality
Leaf	242,841	2.69	25,805	6
Coleoptile	182,785	2.29	21,013	2
Root tip	297,806	2.16	33,619	3
Growth zone	304,934	2.75	35,048	12
Developing grain	239,213	2.46	23,176	6

Following the production of pseudomolecules via reference-guided scaffolding, *LTR_Retriever* identified 608.7 Mbp (61.6%) LTR-RT content in the assembly (assigned and unassigned contigs inclusive). *EDTA* identified 730.1 Mbp of repeat sequences in the pseudomolecules (Table 4, Supplementary File S2b), including 53.07% LTR-RT sequences. A peak in insertion events for both *Gypsy* and *Copia* LTR-RT elements was observed at ~ 0.5 million years ago (MYA; Supplementary Fig. S7). *Gypsy* repeat elements are more abundant than *Copia* elements in the *O. australiensis KR* genome. A third ‘unknown’ LTR-RT superfamily shows a slow increase in copy number over the last four million years (Supplementary Fig. S7).

**Table 4:** Repeat elements in the *O. australiensis* KR scaffolds^51^.

Class	Superfamily	Count	Masked (Mbp)	Masked (%)
LTR-RT	*Copia*	168,433	97.5	11.4
	*Gypsy*	299,854	319.2	37.2
	Unknown	63,847	39.1	4.6
Non-LTR-RT	–	365,794	167.2	19.5
Total	–	897,928	623	72.7

Using the HQ Iso-Seq transcripts and protein sequences from the *Oryza* genus as evidence*, OmicsBox* identified 52,090 genes and 60,325 transcripts in the assembly (Supplementary Files S3c, S4d). *OmicsBox* functionally annotated 35,620 genes and 43,362 transcripts. To check whether our annotation included TE genes, we searched the gene transcripts for known plant TE families^55^. Of our annotated genes 1033 sequences (829 *Gypsy* LTR-RTs, 81 *Copia* LTR-RTs, 4 unknown LINEs, 105 CACTA TIRs, 4 MuDR TIRs, 8 Pong TIRs, and 2 unknown non-LTR-RT repeat elements) were found to be TE genes and removed from the *O. australiensis* gene annotation. After accounting for the presence of TE genes we were left with 51,057 predicted genes, 34,587 functionally annotated genes, and 42,329 functionally annotated transcripts.

### Genotypic diversity and genetic similarity across four *O. australiensis* accessions and within the genus Oryza

**Figure 3:**
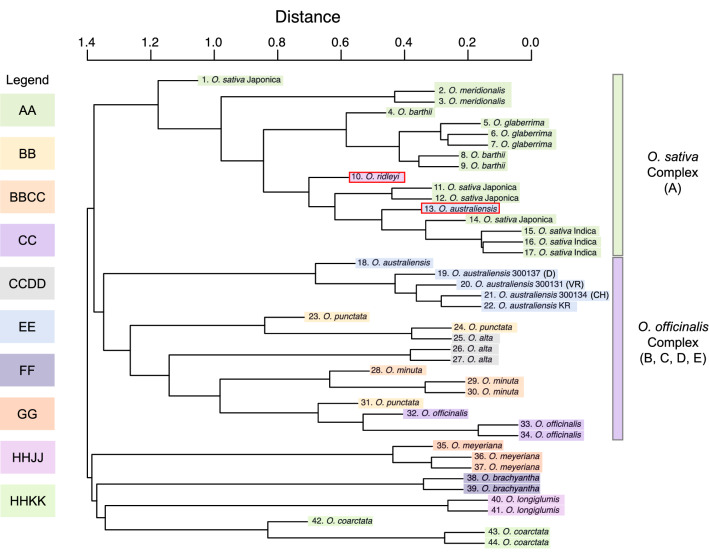
Genetic distance between *Oryza* species/accessions derived from Illumina short read libraries by *kWIP*. Samples are coloured based on the *Oryza* genome clade that they occupy (see inset Legend). *kWIP* clustered samples into the canonical *O. sativa* (AA genome) and *O. officinalis* (BB, CC, BBCC, CCDD, EE genomes) complexes. The *O. australiensis* lineage is divergent from the rest of the *O. officinalis* genome complex, suggesting it harbours lineage specific adaptations that could be explored for stress tolerance. Within the *O. australiensis* lineage, the four genotype samples re-sequenced in the present study (KR, CH, VR, and D) show genetic distances that are correlated with geographic distance. The sample divergence between KR, CH, VR, and D, as well as the other *O. australiensis* accessions shown here suggests that there may be within-species genomic variation that can also be explored for tolerance to stresses (e.g., genotype-specific tolerance to salt stress). Samples that appear with red borders are suspicious as they did not cluster with their corresponding genome clade. This may be due to errors in the sequencing files that were not corrected prior to running *kWIP* or may reflect human error during sample preparation for sequencing.

After mapping the *O. australiensis* KR short reads to the *O. australiensis* KR assembly, we detected ~ 2 million SNVs. In comparison, we detected ~ 2.9 million, ~ 5 million, and ~ 6.4 million SNVs when mapping the CH, D, and VR accession short reads to the *O. australiensis* KR assembly. In the samples we analysed, genome wide SNV number did not increase with increasing geographic distance (Fig. 1). On the other hand, Fig. 3 shows that the CH accession is most closely related to KR, followed by VR, and then D—that is, genetic distance (as determined by *kWIP*) increases with increasing geographic distance (Figs. 1, 3).

Genetic distance between 15 species/accessions (representing the AA, BB, CC, BBCC, CCDD, EE, FF, GG, HHJJ, and HHKK genomes) within the *Oryza* genus was estimated with *kWIP*. A full list of the short read accessions used can be found in Supplementary File S5e. The genetic distances estimated by *kWIP* allowed for the reconstruction of the *Oryza* phylogeny except for two ‘misplaced’ samples (10. *O. ridleyi*, and 13. *O. australiensis*; Fig. 3). We were able to re-construct the canonical *O. sativa* (AA) and the *O. officinalis* (BB, CC, CCDD, EE) genome complexes, and the remaining *Oryza* genomes (FF, GG, HHJJ, HHKK) form a third complex. The *O. australiensis* lineage diverges from the rest of the *O. officinalis* complex members (Fig. 3).

Mapping the *O. australiensis* KR short reads to the *O. sativa* Nipponbare reference genome revealed ~ 2.9 million SNVs between *O. australiensis KR* and *O. sativa* Nipponbare (Supplementary Fig. S8). There are regions on all *O. sativa* chromosomes that, from visual inspection, have low SNV density. Also, the density of SNVs appears to increase at both terminal ends of the *O. sativa* chromosomes, though this may be due to uneven short read mapping density across the *O. sativa* Nipponbare genome (Supplementary Fig. S9).

## Discussion

In this study we report on the first long-read assembly of an *O. australiensis* nuclear genome. Our results indicate that the assembled genome is of high quality and completeness. The assembled pseudomolecules (excluding ChrUn) are, on average, 2.2 times larger than the *O. sativa* Nipponbare reference chromosomes, which is in line with previous observations that *O. australiensis* chromosomes are larger than other *Oryza* species when seen during mitosis^58^. Our scaffolded *O. australiensis* genome assembly (858 Mbp) is smaller than estimated by flow cytometry (965 Mbp^16,58^), however our k-mer based estimation of genome size using *O. australiensis* short reads predicted a genome of 822 Mbp. The discrepancy in size between the genome reported here and the estimated genome size for *O. australiensis*^15,16^ may come about due to irresolvable LTR-RT elements.

After aligning the ONT long reads used to assemble the *O. australiensis* KR contigs to the reference-guided pseudomolecules, we observed unequal coverage distribution on some regions of the pseudomolecules (Supplementary Fig. S4). Most often, coverage was halved in these regions (for example, see Chr4). Having purged the assembly of haplotigs it is unlikely that low coverage results from the presence of homologous maternal and paternal sequences being present in the assembly. Instead, it appears that the low coverage came about due to the relatively high number of small contigs that make up some parts of the pseudomolecules. For example, Chr4, which has the lowest median long read coverage, is composed of the most contigs compared to all other pseudomolecules (apart from ChrUn; Supplementary Fig. S5). The fragmented nature of the contigs that have been used to build these regions of Chr4, and the other pseudomolecules, is characteristic of repetitive genome regions that are typically difficult to assemble. To overcome the limitations imposed by highly repetitive genome regions and, thus, to resolve the sequences therein, it is recommended that future studies employ long reads sufficient for a higher genome coverage.

We found that ~ 74% of the assembly is repeat sequence, of which ~ 55.5–61.6% was identified as LTR-RTs. These estimates are in line with previous estimates (~ 65%) of *O. australiensis* genome repeat content^15^. Stein et al.^1^ report a maximum total repeat content of 49.6% for *O. punctata* (BB genome), and a minimum of 27.2% for *O. meridionalis* (AA genome), thus *O. australiensis* has the highest total repeat content among the few *Oryza* species sequenced to date. We show that there was a relatively recent (~ 0.5 MYA) increase in LTR-RT (*Gypsy* and *Copia*) composition of the *O. australiensis KR* genome, in line with previous observations of *O. australiensis* Bacterial Artificial Chromosome (BAC) library sequences^15^. Similar trends of expansion for these LTR-RT types have been documented for both *O. sativa* indica and *O. sativa* ssp. *japonica* varieties^1^. However, along with a recent expansion of these LTR-RTs, the *O. sativa* genomes have undergone subsequent substantial gene loss, which has played a role in determining the genome size^59^. We also report on the expansion of an unknown LTR-RT family that appears to be in progress in modern-day *O. australiensis* plants, similar to the two *O. sativa* subspecies mentioned above and other *O. australiensis* accessions^15^. Piegu et al.^15^ showed that the LTR-RT element called ‘*Kangourou’*, a low copy number LTR-RT relative to *Gypsy* or *Copia*, had a peak burst time of ~ 2.3 MYA. The magnitude of the copy number burst in the *O. australiensis* genome of *Kangourou* in previous studies using BAC libraries^15^ matches that of the ‘unknown’ LTR-RT element we report here, however our results suggest a much more recent (~ 0.2 MYA) but slower increase in copy number. Other species of wild rice appear to have more ancient LTR-RT family (*Gypsy*, *Copia*, and unknown) expansions. For example, the *Gypsy*, *Copia*, and unknown LTR-RT families of *O. brachyantha* peaked in abundance around 1.8, 2, and 2.5 MYA, respectively^1^. Shenton et al.^13^ show a burst in the *Mutator* MuDR2 family composition of the *O. officinalis* genome complex. Further, *O. meridionalis* seems to have more dated (~ 1–1.5 MYA) expansions and then contractions of the same LTR-RTs relative to *O. australiensis* KR^1^. However, given the differences in the assembly methods and qualities of the various *Oryza* genomes, it is difficult to make direct comparisons of LTR-RT family ages and so these speculations should be viewed with caution.

After accounting for TE genes in the annotation file, we predicted 51,057 genes and functionally annotated 34,587 of those genes. This is in line with previously reported *Oryza* genome annotations. For example, Stein et al.^1^ report a maximum of 38,550 annotated loci for O*. sativa* ssp. *japonica*, and a minimum of 24,208 for *O. brachyantha*. In comparison, Release 7 of the *O. sativa* Nipponbare genome contains 39,045 non-TE annotated genes and 49,066 non-TE gene models^46^. Thus, the number of genes/transcripts annotated in this version of the *O. australiensis KR* genome assembly is comparable to that of the *O. sativa* Nipponbare version 7 genome. This is consistent with the hypothesis that the size of the *O. australiensis* genome is the result of the expansion of LTR-RT elements, rather than gene duplication events^15^. However, similar numbers of coding genes invite speculation about a functional role for expansion of LTR-RT elements. For example, it is conceivable that the especially adverse environments where *O. australiensis* evolved in northern Australia have selected for expansion of particular gene families involved in tolerance to stress.

Comparing the *O. australiensis* KR genome assembly to the *O. sativa* Nipponbare assembly, we identified a number of large structural variations. Recently, Stein et al.^1^ showed an inversion on chromosome two of non-AA rice genomes relative to AA genome rice. The same inversion appears in *Leersia perrieri* and *Brachypodium distachyon* and so it may be a conserved feature in grass genomes, except in rice with the AA genome^1^. This inversion was not detected by reference-guided construction of the *O. australiensis* assembly (Fig. 2), suggesting a need for deeper coverage of this region in future studies. However, we can report 36–45× coverage at the putative inversion junction in the *O. australiensis* KR assembly, and we show that the putative SV sits well within the boundaries of a single contig. Hence, we suggest that this region of the *O. australiensis* KR assembly is not the result of misassembly. The possibility remains that it is an artefact of reference-biased scaffolding, however we found that the inversion is missing in the *O. australiensis* assembly even after using a BB rice genome (*O. punctata*) to order and orient the contigs (data not shown), so this is unlikely. The SV reported by Stein et al.^1^ in non-AA rice genomes may truly be absent from the *O. australiensis* KR genome. One hypothesis for its absence is hybridisation between the co-occurring AA-genome species (*O. meridionalis* and *O. rufipogon*) and *O. australiensis*, thus eliminating the Chr2 SV in the *O. australiensis* KR genome. However, given the difficulty in producing viable progeny from artificial crosses between the AA genome species and *O. australiensis*^60^, we find this to be an improbable explanation for the observed results. Thus, in the absence of further evidence, all structural variations should be viewed with caution.

Given that the 21 large SVs we observed do not appear to be the result of either scaffolding or assembly artefacts, if these SVs prove to be real, they highlight considerable differences in the structure of the *O. australiensis KR* assembly and *O. sativa*. These SVs, and the substantial repeat content of the wild rice genome, would offer insight into why crossing the AA genome *O. sativa* and the EE genome *O. australiensis* is difficult and why successful crosses have abnormal chromosomal arrangements and the resulting progeny are sterile^61,62^. To validate the putative SVs, future scaffolding attempts could make use of other *Oryza* species for reference-guided scaffolding (e.g., the *O. officinalis* assembly may be a better candidate for scaffolding than the *O. sativa* Nipponbare assembly as *O. australiensis* is a member of the *O. officinalis* complex). However, this would still present biases in contig ordering and orientation. Therefore, we recommend that future scaffolding attempts either make use of pre-existing *O. australiensis* BAC libraries and FingerPrinted Contigs (FPC) to order and orient the contigs^1,13^ or use Hi-C technology to generate new data for *O. australiensis*. Given the still experimental nature of Hi-C and difficulties in obtaining high coverage of linked reads these efforts may require significant investment.

Along with structural variations in the *O. australiensis KR* genome relative to *O. sativa* Nipponbare, we also uncovered many SNVs (Supplementary Fig. S8). Interestingly, there were more SNVs between some *O. australiensis* genotypes and *O. australiensis* KR than there were between *O. sativa* and *O. australiensis* KR based on short read mapping. The density of SNVs increases at the terminal regions of the *O. sativa* chromosomes. Conversely, there is a region on each chromosome where the density of SNVs is low. These regions may represent the centromeric repeat elements of the chromosome (Supplementary Fig. S8). While centromeric regions have a common functional role in organising cell division, their sequences can be diverse between species. However, in the case of rice and other cereals, they are highly conserved^63,64^. For example, Gao et al.^65^ showed that *O. australiensis* and *O. sativa* share similar centromeric retrotransposon repeat (CRR) sequences even though other rice species such as *O. brachyantha* have unique CRR profiles. This helps explain why sequence similarity between *O. australiensis KR* and *O. sativa* at centromeric regions is higher than at other regions of the genome.

Interestingly, when comparing four genotypes of *O. australiensis* that had been documented for their contrasting salt tolerance^20^, we found that SNV density was not well correlated with the geographical distance between each of the populations (Fig. 1). For example, VR had at least 1.5 million more SNVs than the other two genotypes (when compared with KR), yet it was collected from a site far closer to Keep River than the D genotype. We speculate that SNV densities may be more related to the diversity of alleles required for adaptation to stresses than physical separation between populations. For example ‘relative salt tolerance’^20^ (Fig. 1) appeared to correlate better with the abundance of SNV abundance than simple distance between populations.

Contrary to the SNV data, the genetic distances estimated by *kWIP* *do* reflect the geographic distance between the *O. australiensis* genotypes (Fig. 3). That is, populations of *O. australiensis* genotypes that occur closer to each other in space are more genetically similar than genotypes that occur further apart. This is expected as *O. australiensis* is a mostly inbreeding species (~ 3% outcrossing), so gene flow and introgressions between genotypes should be low. Thus, we assume that these populations of *O. australiensis* are reproductively isolated and, as such, their genomes are evolving independently. If this is true, we expect to see local adaptation in these genotypes^66^, a hypothesis consistent with the findings of Yichie et al.^20^.

The genetic-distance tree generated by *kWIP* follows previously published *Oryza* phylogenies^13^. Importantly, the *O. australiensis* lineage diverges from the rest of the *O. officinalis* complex members (the B, C, and D genomes) suggesting that we can expect to see lineage specific adaptation in the *O. australiensis* genome. Given that this species occupies a diverse yet adverse suite of environmental niches, the genomic variation that underpins the divergence of the *O. australiensis* lineage may be associated with tolerance to multiple abiotic and biotic stresses. The functionally annotated genome that we present for *O. australiensis* KR here is therefore a resource that can be used to explore this genomic variation to uncover genes involved in stress tolerance.

## Conclusions

Here we present the first reference-quality genome of *O. australiensis KR*—a wild species of rice native to the northern regions of Australia. While *O. australiensis* co-occurs with other *Oryza* species, it is genetically and phenotypically unique among the *Oryza* genus and is the sole member of the EE genome clade^67^. It demonstrates unrivalled tolerance to extreme environmental conditions, including heat, drought and soil salinity^3,18,21,68^. The leaf anatomy and associated photosynthetic efficiency of *O. australiensis* are also unique traits^22^ and its grain has distinct pigmentation and starch composition^23,24^. Such traits make it an important genetic resource in the *Oryza* genus, as recognised formally by the OMAP and IOMAP^11,12^. However, no group has successfully assembled the nuclear genome for this species until now. It is expected that this genomic resource will enable phenomics in wild cereal relatives and lead to the selection of useful traits for the improvement of domestic rice cultivars, and in the breeding and domestication of *O. australiensis* itself. For example, given that we now know the positions of genes in the *O. australiensis* KR assembly, one can use the assembly alongside short reads from diverse *O. australiensis* accessions to identify SNVs in genes associated with tolerance in accessions from adverse environments. Similarly, with further Iso-Seq data one could identify novel transcript isoforms across different *O. australiensis* accessions under multiple stresses. Furthermore, given the role that gene regulation plays in response to stress, the *O. australiensis* KR assembly will uncover promoters of genes of interest. This work could extend to the use of long read data (either PacBio or ONT reads) to investigate the response of the epigenome to abiotic stresses. Finally, an expanded collection of *O. australiensis* accessions with contrasting phenotypes under extreme conditions will facilitate the identification of markers for stress tolerance via strategic inclusion of tolerant accessions to make mapping populations.

## Supplementary Information

Supplementary Figures.

## Data Availability

This Whole Genome project (i.e., the *Canu*-generated purged contigs reported here) has been deposited at DDBJ/ENA/GenBank under the accession JAIFGZ000000000. The version described in this paper is version JAIFGZ010000000. All long- and short-read data associated with the reported genome assembly have been deposited at the Sequence Read Archive and GenBank in NCBI under BioProject PRJNA743927. The AGP file required to construct the pseudomolecules from the *Canu* contigs, the annotation for protein-coding genes in the contigs, the annotation for repeat elements in the scaffolds, and the annotation for protein-coding genes in the scaffolds (all GFF3 files) have been provided as Supplementary Files and can be accessed via figshare: 10.6084/m9.figshare.c.5875592.v2.

## Abbreviations

AGP: A golden path
ANU: Australian National University
BAM: Binary Alignment Map
BCF: Binary variant call format
BLAST: Basic local alignment search tool
bp: Base pairs
BUSCO: Benchmarking universal single-copy orthologs
Chr: Chromosome
ChrUn: Chromosome unassigned
DVC(R): Deputy Vice Chancellor (Research)
EDTA: Ethylenediaminetetraacetic acid
EDTA: Extensive de novo TE annotator
GFF3: General Feature Format 3
GO: Gene Ontology
HPC: High performance computer
HQ: High quality
IGV: Integrative genomics viewer
IOMAP: International *Oryza* Map Alignment Project
Iso-Seq: Isoform sequencing
Kbp: Kilo base pairs
LAI: LTR assembly index
LQ: Low quality
LTR: Long terminal repeat
RT: Retrotransposon
Mbp: Mega base pairs
MYA: Million years ago
NCBI: National Center for Biotechnology Information
NSW: New South Wales
OMAP: *Oryza* Map Alignment Project
ONT: Oxford Nanopore Technologies
PAF: Pairwise mApping format
PEG: Polyethylene glycol
PIF: P Instability factor
PVP: Polyvinylpyrrolidone
RIN: RNA integrity number
RMBlast: RepeatMasker compatible NCBI BLAST suite
SAM: Sequence Alignment Map
SNV: Single nucleotide variation
SV: Structural variation
TIR: Terminal inverted repeat
VCF: Variant call format
