# Supplementary material for: The first long-read nuclear genome assembly of Oryza australiensis, a wild rice from northern Australia
Source: Sci Rep. 2022 Jun 25;12:10823. doi: 10.1038/s41598-022-14893-5 (PMC9233661; doi:10.1038/s41598-022-14893-5)
Supplement: Supplementary file 1 — Supplementary Figures. [file 41598_2022_14893_MOESM1_ESM.pdf]

Supplementary Material

All supplementary material can be found at:
https://doi.org/10.6084/m9.figshare.c.5875592.v2
Please access difficult-to-see figures at the link above for high resolution versions of the figures.

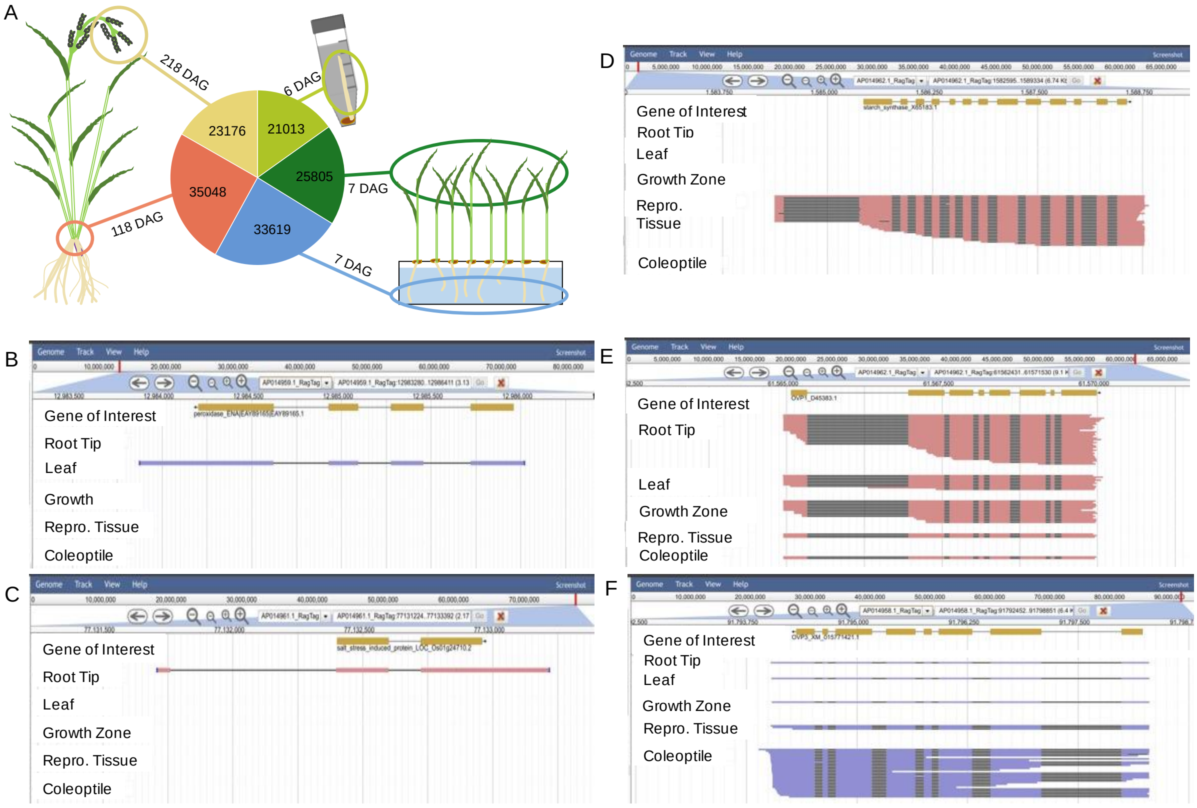

Supplementary Figure S1. High-quality transcripts derived from Iso-Seq data. (A) total abundance of HQ transcripts for five *O. australiensis* Keep River tissues (red = reproductive tissue [rachillae, developing seed, and pollen], dark blue = 7-day old leaves, light blue = 7-day old root tips, green = 6-day old coleoptiles, orange = growth zones). (B-F) tissue-specific detection of select transcripts (peroxidase [EAY89165.1], salt-stress-induced protein [LOC_Os01g24710.2], starch synthase [X65183.1], *Oryza* vacuolar pyrophosphatase 1 [OVP1; D45383.1], and *Oryza* vacuolar pyrophosphatase 3 [OVP3; XM_015771421.1], respectively) involved in response to several abiotic stressors.

Figure too large to display. Please access the figure at link provided above.

Supplementary Figure S2. Long-read coverage of the *O. australiensis* Keep River contigs. Long reads used to assemble the contigs were mapped to the contigs and the resulting indexed BAM file was used by JVarkit's wgscoverageplotter tool to find genome coverage. The green line represents the average coverage, and the red line represents the median coverage. Note: not all contigs are shown here.

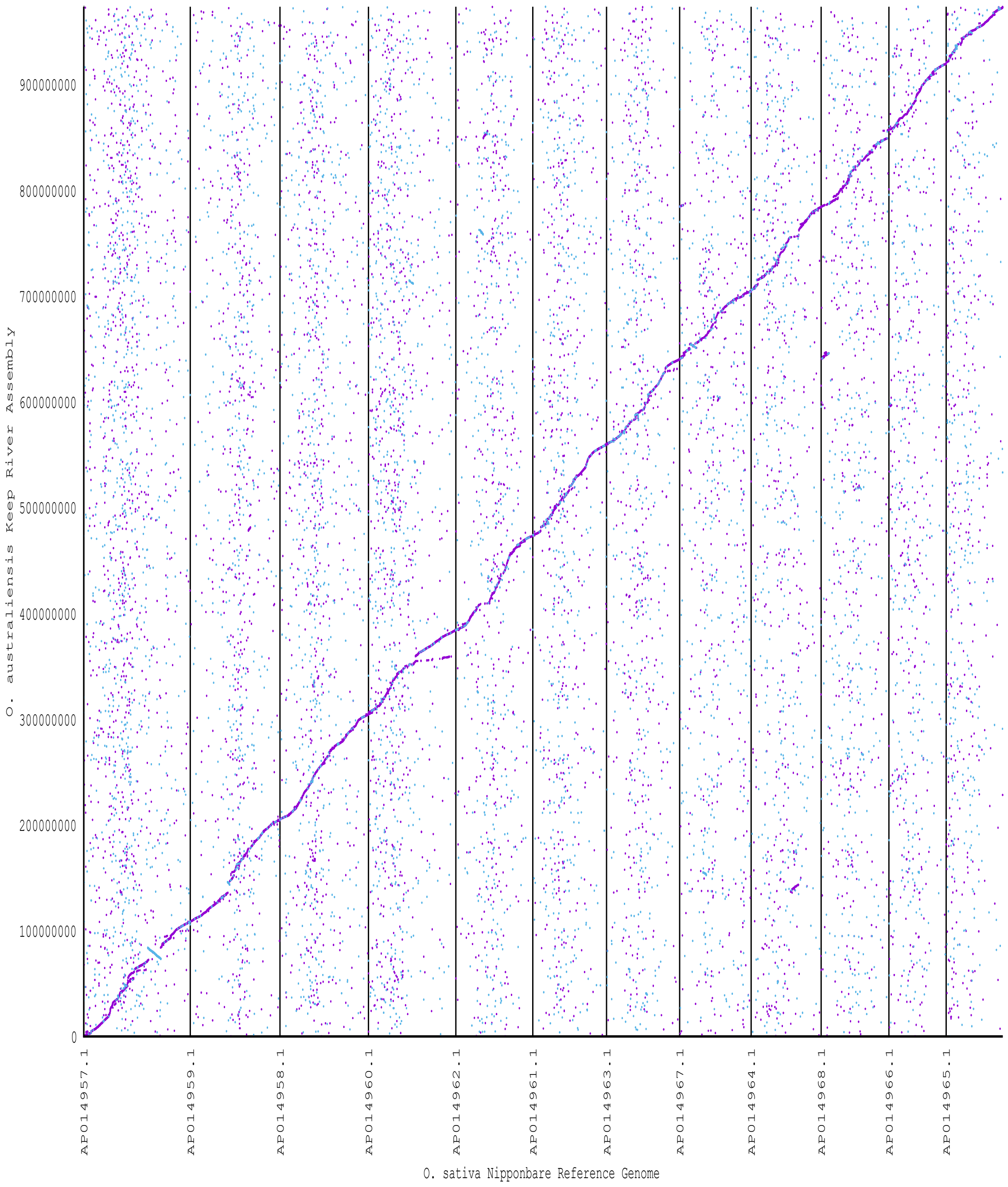

Supplementary Figure S3. Whole genome alignment of *O. australiensis* Keep River contigs and *O. sativa* Nipponbare chromosomes. Shows a genome-genome alignment between the *O. australiensis* Keep River contigs described in this paper and the *O. sativa* Nipponbare reference genome. Contigs were mapped to the *O. sativa* Nipponbare chromosomes using minimap2 and the resulting alignment was visualised, using MashMap. The X-axis shows the *O. sativa* chromosomes, while the Y-axis shows the *O. australiensis* contigs, expressed in Mbp.

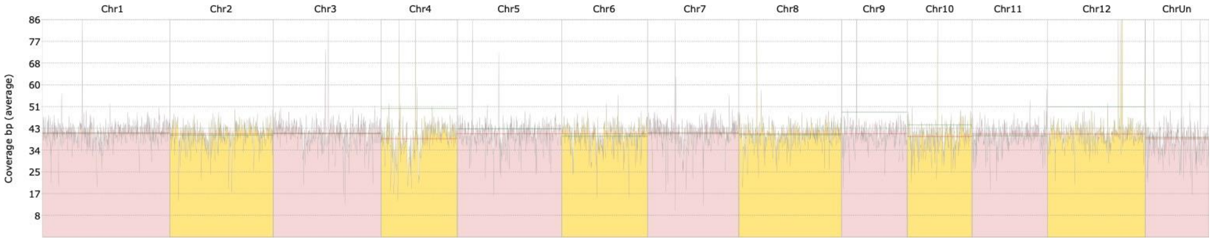

Supplementary Figure S4. Long-read coverage of the *O. australiensis* Keep River pseudomolecules. Shows a long-read coverage plot for the *O. australiensis* Keep River pseudomolecules. Long reads used to assemble the contigs were mapped to the pseudomolecules and the resulting indexed BAM file was used by JVarkit's wgscoverageplotter tool. The green line represents the average coverage, and the red line represents the median coverage.

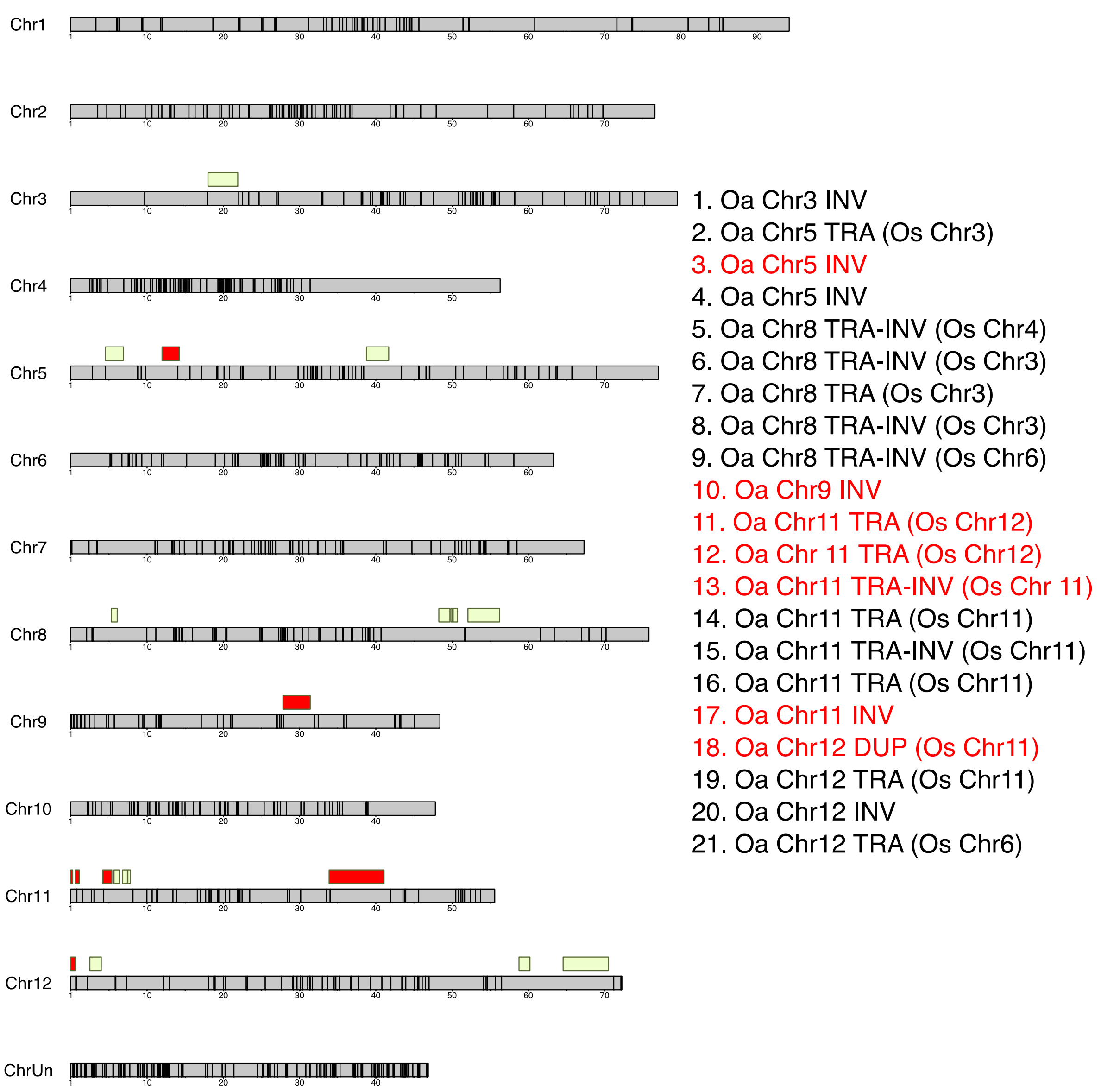

Supplementary Figure S5. Location of contig-contig boundaries, and assessment of, the structural variants (SVs) detected in *O. australiensis* KR pseudomolecules. Black lines indicate the boundaries between contigs, which are made up of 100 Ns. The light green bands represent the major SVs (inversions, translocations, and duplications) relative to *O. sativa* Nipponbare. SV coordinates (± 10 Kbp) were manually curated using an interactive *dotPlotly* plot of the whole-genome alignment between *O. australiensis* and *O. sativa* and the visualised using *karyoploteR* in the R environment. SVs (± 10 Kbp) that are wholly contained within a contig are likely to be real and not artefacts of incorrect reference-guided contig orientation. SVs shown in red indicate those that cross a contig-contig boundary. The manually curated list of SVs (inset) reports SVs as they occur in numerical order. The scale bars beneath each scaffold show the size of the scaffolds in Mbp.

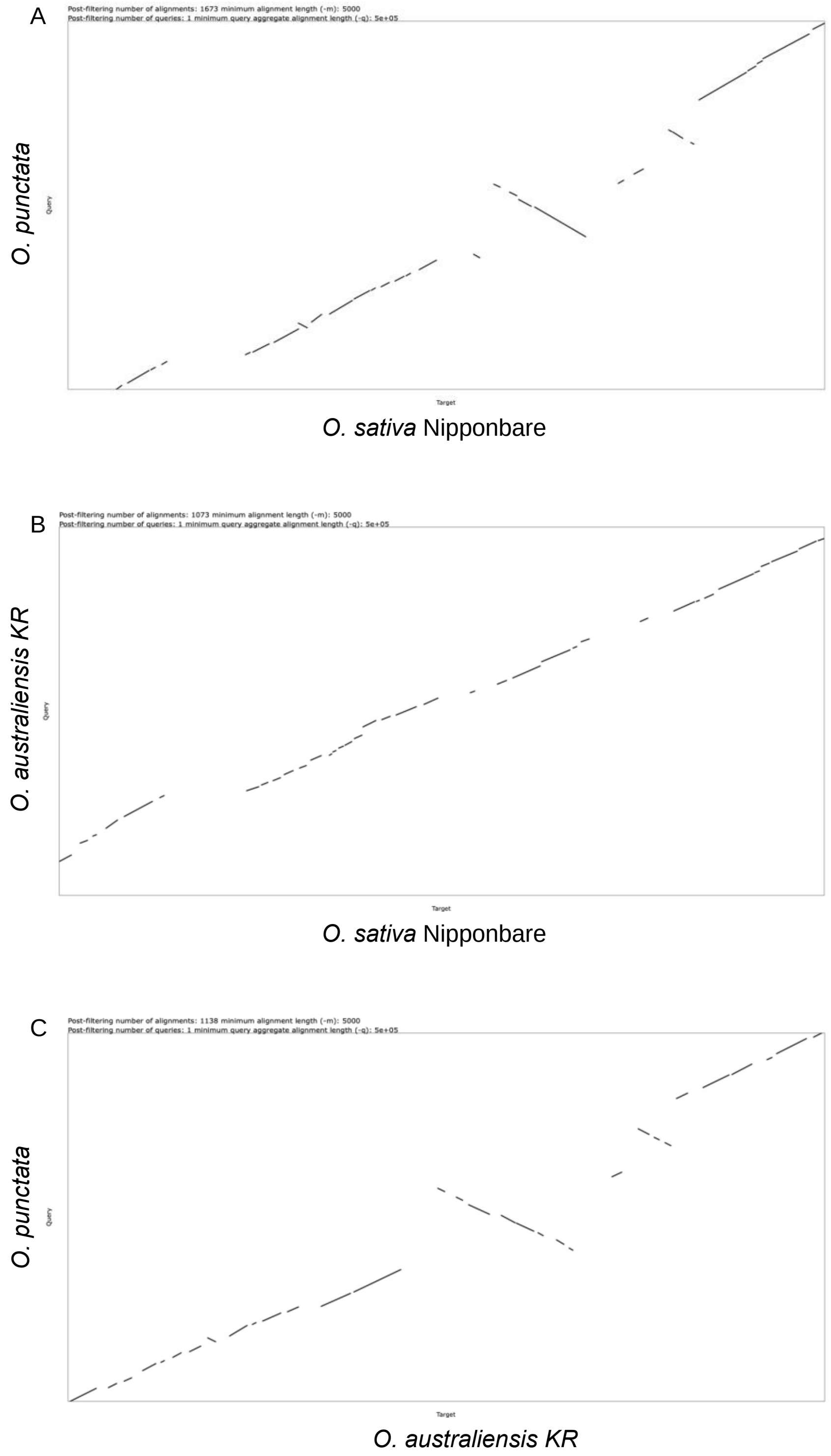

Supplementary Figure S6. Inspection of Chr2 for a previously reported inversion. (A) Alignment between *O. sativa* Nipponbare Chr2:34-35 Mbp and *O. punctata* Chr2 (CM002489.2). This alignment shows an inversion on *O. punctata* Chr2 relative to *O. sativa* Chr2. (B) The alignment between *O. sativa* Nipponbare Chr2 and *O. australiensis* KR Chr2 does not show the canonical non-AA rice Chr2 inversion. (C) The alignment between *O. australiensis* KR Chr2 and *O. punctata* Chr2 shows the same inversion seen in (A). Thus, we do not observe the previously reported Chr2 inversion in the *O. australiensis* KR assembly.

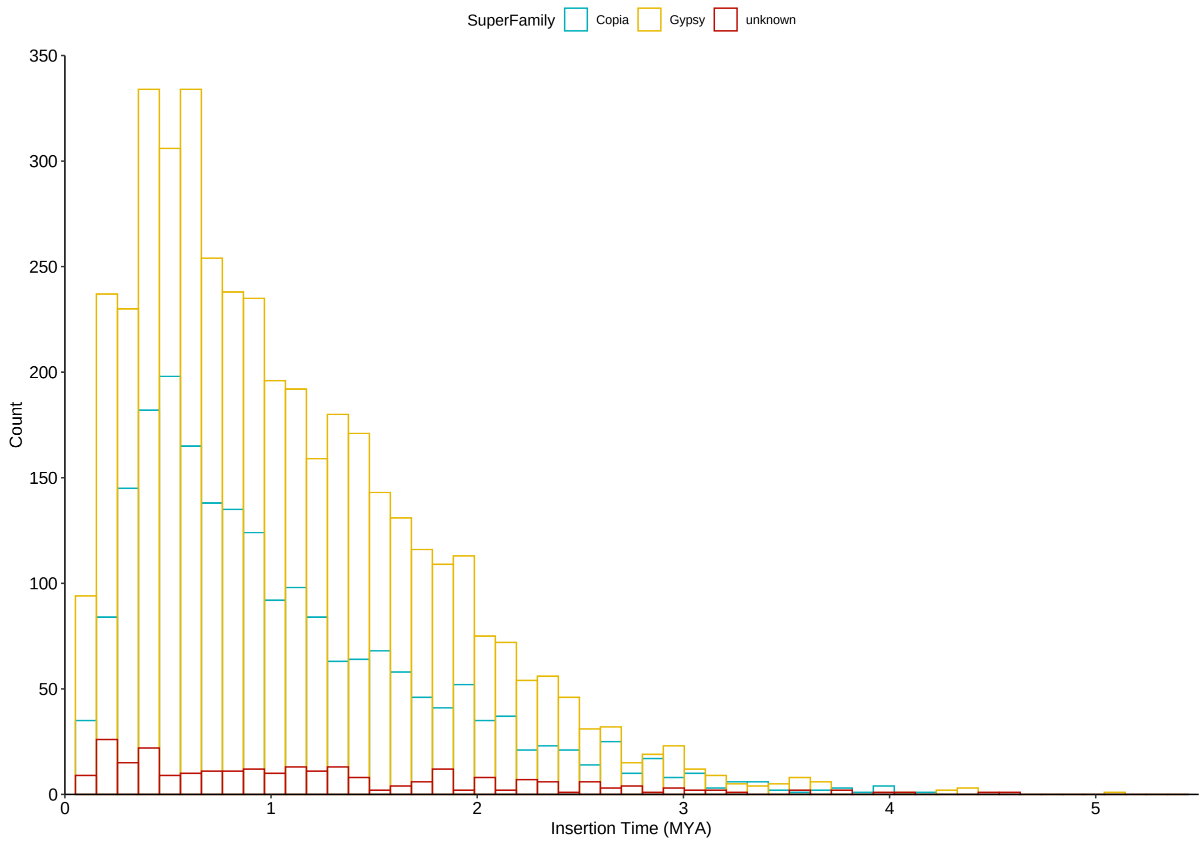

Supplementary Figure S7. Insertion times for the major LTR-RT superfamilies identified in the scaffolded *O. australiensis* nuclear genome assembly (including ChrUn) by *LTR_Retriever*.

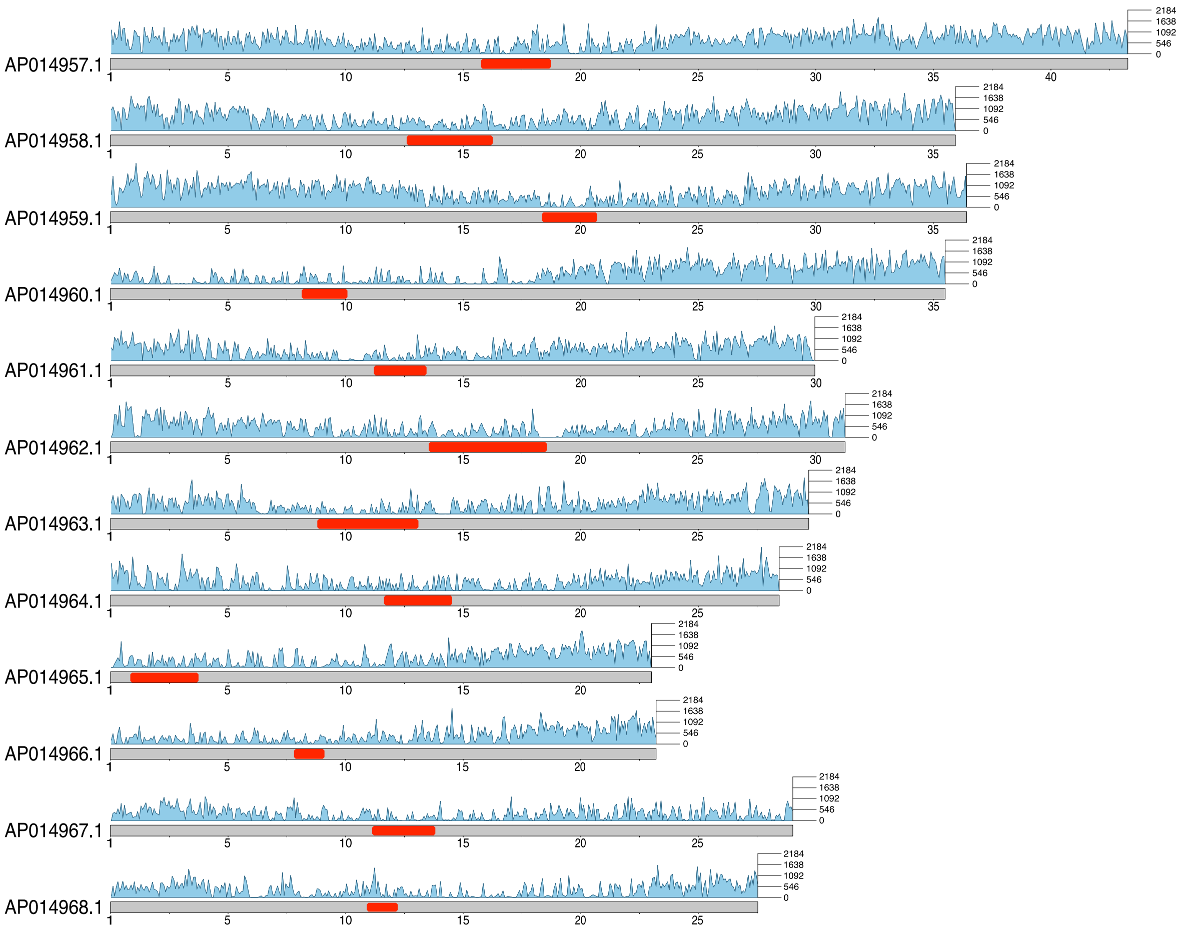

Supplementary Figure S8. SNVs identified between the assembled *O. australiensis* Keep River genome (blue) and *O. sativa* Nipponbare reference genome (grey rectangles). The *O. sativa* chromosomes appear in numerical order from top to bottom. Red boxes indicated location of centromeric regions on the *O. sativa* chromosomes as reported by Mizuno et al.[57]. SNVs were detected by mapping *O. australiensis* Keep River short reads to the *O. sativa* Nipponbare genome and using mpileup to call SNVs. Chromosomes appear in order from 1 to 12.

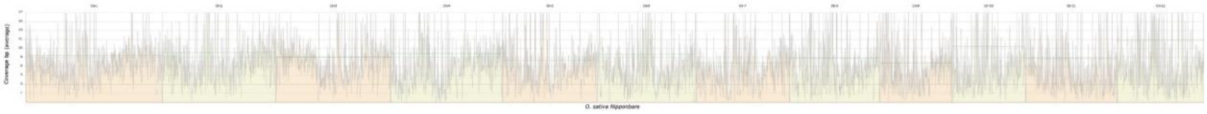

Supplementary Figure S9. Short-read coverage plot. *O. australiensis* KR Illumina short reads were mapped to O. sativa Nipponbare chromosomes and the resulting indexed BAM file was used by JVarkit's wgscoverageplotter tool to plot per-base coverage. The green line represents the average coverage for each chromosome. O. australiensis KR short read coverage is not even across each chromosome, which may affect analyses of SNV density.

Supplementary File S1a. Contig-to-scaffold AGP file generated by *Ragtag*. This AGP file is used to constructs scaffolds (pseudomolecules) from the primary contigs contained at NCBI under the accession number: JAIFGZ000000000. Canu assembled contigs were purged using Purge Haplotigs, and the resulting KEEP and REPEAT contigs were uploaded to NCBI.

Supplementary File S2b. EDTA GFF3 annotation of repeat sequences in the scaffolds. This file contains the repeat elements identified by Extensive de novo TE Annotator (EDTA) in the *O. australiensis* Keep River genome assembly. It is in the format of a GFF3 and maps to the assembly when scaffolded using the supplied AGP file (Supplementary File S1a).

Supplementary File S3c. Omicsbox GFF3 annotation of protein coding genes in the contigs. This file contains functionally annotated protein-coding genes for the O. australiensis Keep River assembly in Contig form at NCBI: JAIFGZ000000000.

Supplementary File S4d. Omicsbox GFF3 annotation of protein coding genes in the scaffolds. This file contains information about functionally-annotated protein-coding genes for the scaffolded version of the *O. australiensis* Keep River genome assembly stored at NCBI in contig form: JAIFGZ000000000. Scaffolds can be obtained using the AGP file provided in this repository to order and orient the contigs. Note: contigs that could not be placed within scaffolds were annotated as individual contigs, not as ChrUn. Therefore, when using the AGP file to build scaffolds from the contigs, ChrUn lines should be excluded. Also note: this file contains annotations for the discarded contigs (REPEAT, JUNK and HAPLOTIG).

Supplementary File S5e. *Oryza* accessions for KWIP. Contains information about the accessions used for estimates of genetic similarity by kWIP, including four accessions sequenced as part of the present study.
